# Two Functional *TP53* Genetic Variants and Predisposition to Keloid Scarring in Caucasians

**DOI:** 10.1155/2019/6179063

**Published:** 2019-11-13

**Authors:** Andrzej Dmytrzak, Agnieszka Boroń, Beata Łoniewska, Klaudyna Lewandowska, Iwona Gorący, Mariusz Kaczmarczyk, Andrzej Ciechanowicz

**Affiliations:** ^1^Aesthetic Med Andrzej Dmytrzak Prywatne Centrum Chirurgii Plastycznej i Rekonstrukcyjnej, ul. Niedzialkowskiego 47, 71-403 Szczecin, Poland; ^2^Department of Clinical and Molecular Biochemistry, Pomeranian Medical University, al. Powstancow Wlkp. 72, 70-111 Szczecin, Poland; ^3^Department of Neonatal Diseases, Pomeranian Medical University, ul. Powstancow Wlkp. 72, 70-111 Szczecin, Poland

## Abstract

**Introduction:**

Keloid is defined as a benign proliferative scar that grows beyond the confines of the original insult to the skin, invading into adjacent normal tissue. The pathogenesis of keloid is complex, and many evidences suggest the influence of genetic factors, among them the polymorphisms of the *TP53* gene encoding tumor protein p53.

**Objective:**

To investigate the association of rs1042522 (c.215G>C, p.Arg72Pro) and rs17878362 (16-bp insertion/duplication in intron 3) variants, two most frequently analyzed *TP53* functional polymorphisms and the risk of keloid in Polish patients.

**Materials and Methods:**

The rs1042522 and rs17878362 polymorphisms were identified by sequencing genomic DNA extracted from peripheral blood leukocytes of 86 keloid patients and from cordial blood leukocytes of 100 newborn infants consisting control group.

**Results:**

The rs1042522 and rs17878362 *TP53* genotype distributions both in keloid patients and in the control group conformed to the expected Hardy–Weinberg equilibrium. No significant differences in the distribution of rs1042522 and rs17878362 *TP53* alleles or genotypes have been found between keloid patients and newborn controls. There is tight, but not complete, linkage disequilibrium between rs1042522 and rs17878362 *TP53* polymorphisms (*D*′ = 0.667, *r* = 0.448, and *p*=0). No significant differences in the distribution of rs1042522 and rs17878362 *TP53* haplotypes or diplotypes have been found between keloid patients and newborn controls.

**Conclusions:**

Our results suggest the lack of association of rs1042522 and rs17878362 *TP53* polymorphisms and their haplotypes or diplotypes with the susceptibility to keloid scarring in Polish patients.

## 1. Introduction

Keloid scar is defined as a dermal benign fibro-proliferative growth that extends outside the original wound and invades the adjacent dermal tissue due to extensive production of extracellular matrix, especially collagen, which is caused by over expression of cytokines and growth factors. Although many attempts were made to understand the exact pathophysiology and the molecular abnormalities, the pathogenesis of the keloid scar is yet to be determined [1]. The mechanisms of keloid formation include, among others, decreased apoptotic activity, alterations in growth factors, impaired collagen turnover, as well as immunological and genetic contributions [2, 3]. Recently, Glass pointed out either keloid-linked chromosomal loci or candidate genes for keloid, among the latter *TP53* [4].

*TP53* is a tumor suppressor gene located on chromosome 17p13.1 that encodes protein p53. The tumor protein p53 binds directly to DNA and participates in the regulation of cell cycle checkpoints, DNA repair, and apoptosis and regulates the repair process in response to damaging factors, including chemicals, radiation, and ultraviolet rays from sunlight [5]. It has been reported that rs1042522 (c.215G>C, p.Arg72Pro) and rs17878362 (16-bp insertion/duplication in intron 3), two most frequently analyzed *TP53* polymorphisms, may affect either the function of the p53 protein or its mRNA expression [6–8].

The results of meta-analysis based on 359 keloid cases and 493 healthy controls revealed no association between germinal p.Arg72Pro mutation and susceptibility to keloid in the Chinese population [9]. To the best of our knowledge in contrast to rs1042522, to date no study for association of intronic rs17878362 *TP53* polymorphism with keloid scarring has been carried out.

Therefore, the aim of our study was to analyze the association of rs1042522 and rs17878362 and their haplotypes with the predisposition to keloid in Caucasians.

## 2. Materials and Methods

The study group consisted of 86 consecutive patients with keloid (aged from 18 to 70 years old), who were treated with surgical excision in the Aesthetic Med, Szczecin, Poland. Characteristics of the studied patients are shown in Supplemental [Supplementary-material supplementary-material-1]. The control group consisted of 100 healthy, full-term newborns (52 males and 48 females) randomly chosen from the Newborn DNA Repository at the Department of Clinical and Molecular Biochemistry at the Pomeranian Medical University in Szczecin. All children were breast-fed and free of medication. Twins and infants of mothers with keloid scarring, preeclampsia, hypertension of any cause, diabetes, history of illicit substance use, or antenatal steroid therapy were excluded. Other exclusion criteria were congenital infection, intrauterine growth restriction (i.e., below the 10th percentile birth mass, length, or head circumference), chromosomal aberrations, or congenital malformations. All patients in study and control groups were Poles of European descent. The study was conducted in accordance with the Declaration of Helsinki and was approved by the local bioethics committee at the Pomeranian Medical University in Szczecin. Patient informed consent for cases and parental informed consent for newborn controls were obtained.

Genomic DNA was extracted either from peripheral blood leukocytes (keloid patients) or from cordial blood leukocytes (newborn infants) using a commercially available DNA isolation kit (QIAamp Blood DNA Mini Kit, QIAGEN, Germany). Amplification of the 540-bp *TP53* sequence including rs1042522 and rs17878362 was performed by using PCR with 5′-AACCCCAGCCCCCTAGCAGAGACC-3′ as the forward primer and 5′-GGGGATACGG CCAGGCATTGAAGT-3′ as the reverse primer. Subsequently, PCR amplification products were purified using Exonuclease I and FastAP Thermosensitive Alkaline Phosphatase (ThermoFisher Scientific Inc., Waltham, MA, USA) according to manufacturer procedures. Sequencing of the products used BigDye® Terminator v3.1 Cycle Sequencing Kits (Applied Biosystems, Life Technologies Polska, Warsaw, Poland). Electrophoresis and analysis were performed according to manufacturer procedures using an ABI PRISM 3100-Avant machine (Data Collection Software v2.0, Sequencing Analysis Software v5.4; Applied Biosystems).

Possible divergence of rs1042522 and rs17878362 *TP53* genotype frequencies from the Hardy–Weinberg equilibrium was assessed using a *χ*^2^ test. The association between a pair of analyzed *loci* (linkage disequilibrium, LD) was tested using the *χ*^2^ test with the parameter *D*′ and correlation coefficient *r*. The Hardy–Weinberg equilibrium and LD were analyzed using the “Genetics” package. Frequency differences in frequencies of genotypes, alleles, haplotypes, or diplotypes between groups were tested for statistical significance using the *χ*^2^ test or Fisher's exact test, if necessary. Genotype frequencies between groups were then compared by univariate logistic regression in additive, dominant, or recessive mode of inheritance of the risk allele.

The “haplo.score” and “haplo.glm” functions from the “haplo.stats” package were applied to test the effect of haplotypes. A positive Hap.Score value implies that the haplotype occurs more frequently in the keloid patients than control subjects, whereas a negative Hap.Score indicates that the haplotype occurs more frequently in control subjects. “Genetics” and “haplo.stats” are packages for R a free software environment for statistical analysis (ver. 2.11.1, R Foundation for Statistical Computing, Vienna, Austria, http://www.R-project.org). The remaining calculations were performed using a data analysis software system (Dell Statistica, version 13. Dell Inc. 2016, http://software.dell.com). A two-tailed *p* < 0.05 was considered statistically significant.

## 3. Results

The rs1042522 and rs17878362 *TP53* genotype distributions both in keloid patients and in the control group conformed to the expected Hardy–Weinberg equilibrium (*p*=0.709 and *p*=0.864 or *p*=0.952 and *p*=0.425, respectively). No significant differences in the distribution of rs1042522 and rs17878362 *TP53* alleles or genotypes have been found between male newborn controls and female ones (*p*=0.773 or *p*=0.525 for alleles and *p*=0.961 or *p*=0.427 for genotypes, respectively). No significant differences in the distribution of rs1042522 and rs17878362 *TP53* alleles or genotypes have been found between keloid patients and newborn controls (*p*=0.690 or *p*=0.496 for alleles and *p*=0.184 or *p*=0.718 for genotypes, respectively). Univariate logistic regression revealed no significant association between rs1042522 and rs17878362 *TP53* polymorphisms and predisposition to keloid in additive mode of inheritance of the risk allele (C allele of rs1042522 or A2 allele of rs17878362, respectively) (Table 1) as well as in dominant (*p*=0.107 for rs1042522 or *p*=0.440 for rs17878362, respectively) or in recessive (*p*=0.206 for rs1042522 or not available for rs17878362, respectively) mode of inheritance of the risk allele.

There is tight, but not complete, linkage disequilibrium between rs1042522 and rs17878362 *TP53* polymorphisms (*D*′ = 0.667, *r* = 0.448, *p*=0). No significant association of *TP53* haplotypes and the risk of keloid scarring have been found using both global score and haplotype-specific statistics (Table 2). There are also no significant differences in frequency distribution of *TP53* diplotypes between the keloid patients and control group by comparing every diplotype to the reference one (D1) (Table 3).

## 4. Discussion

Somatic mutations in the *TP53* gene encoding tumor protein p53 are one of the most common genetic abnormalities associated with human cancer and have been implicated as causal events in up to 50% of all human malignancies. Germline *TP53* mutations also increase the risk of numerous neoplasm types, including breast cancer, leukemia, sarcomas, and central nervous system tumors [5].

The rs1042522 is a G to C transversion at the second position of codon 72 in exon 4 leading to substitution of arginine (CGC) by proline (CCC) in p53 polypeptide chain (c.215G>C, p.Arg72Pro). The transversion is located in the N-terminal proline-rich domain (residues 64–92) of the protein required for the growth suppression and apoptosis mediated by the p53 [10]. It has been reported that the p.Arg72 p53 variant induces apoptosis with faster kinetics and suppresses transformation more efficiently than the p.Pro72 one [6]. In addition, Dumont et al. indicated that the higher efficiency of p.Arg72 variant in triggering cellular apoptosis is due to its greater ability to localize to the mitochondria [7].

In 2005, Zhuo et al., using polymerase chain reaction-reverse dot blot (PCR-RDB) and DNA direct sequencing analyzed Chinese subjects (15 patients with keloid and 15 healthy controls) and revealed that the frequency of both p.Pro72 *TP53* allele and p.Pro72 *TP53* homozygous genotype in keloid patients was significantly higher than that in the controls [11]. In contrast, Wang et al. also reported in 2005 that p.Arg72 *TP53* homozygous genotype in Japanese subjects is associated with the risk for the piercing-induced ear-lobe keloid [12]. Further studies performed exclusively in Chinese patients also yielded conflicting results [13–17].

To the best of our knowledge, our study is a first report about rs1042522 *TP53* polymorphism and the predisposition to keloid in subjects of European descent (Poles). The frequency of the minor c.215C (p.Pro72) allele equal 28.5% in our controls was close to values from the 1000 Genomes Project (https://www.ncbi.nlm.nih.gov/variation/tools/1000genomes/) in other European populations, which ranged from 24.2% in CEU (Utah residents with Northern and Western European ancestry) to 30.8% in British individuals in England and Scotland (GBR). The prevalence of p.Pro72 *TP53* allele in Poles is also very close to the variant frequencies found in the Czechs and Slovaks, our nearest neighbors of Slavic origin (Czechs 29.1% or 29.4% and Slovacks 25.4%, respectively) [18–20]. We have found no significant differences in the frequency distribution of both rs1042522 *TP53* alleles and genotypes between keloid patients and newborn controls. On the other hand, the frequency of minor (p.Pro72) allele in keloid patients was 8% lower as compared with control subjects (20% versus 28%, respectively). Also, Liu [15] and Yan et al. [16] reported of 8% lower frequency of p.Pro72 *TP53* allele in Chinese keloid patients as compared with healthy controls. However, it is noteworthy, that the frequency of this allele in Chinese, both in the study (40% or 41%) and control groups (48% or 498%) [15, 16], was significantly higher compared to our data from Caucasians.

The results of meta-analysis based on six studies [11, 13–17] did not confirm the association between rs1042522 *TP53* polymorphism and susceptibility to keloids in Chinese patients [9]. However, subsequent subgroup analysis in regard to genotyping method (PCR-reverse dot blot *versus* PCR-RFLP) revealed contradictory results. The p.Pro72 *TP53* allele was associated with the predisposition to keloid scarring only in patients genotyped by using PCR-reverse dot blot [11, 13–15]. In contrast, the association between p.Arg72 *TP53* allele and predisposition to keloid has been found in patients analyzed by using PCR-RFLP [16, 17]. And although the authors of meta-analysis argue that different detection methods may be the source of the above heterogeneity, we suppose that the major cause of it is rather population bias evidenced in the 1000 Genomes Project (1KG Project). The p.Pro72 *TP53* frequency in CHB (Han Chinese in Bejing), CHS (Southern Han Chinese), and CDX (Chinese Dai in Xishuangbanna) was 45.1%, 40.0%, and 47.3%, respectively. It is also worth emphasizing that the highest p.Pro72 *TP53* frequency was observed in populations of Sub-Saharan Africa (63.9% or 68.2% in Nigeria, 70/8% in Gambia, or even 74.7% in Kenya). And despite both these results of 1KG Project and some previous studies [21, 22] strongly supported the hypothesis that the p.Pro72 *TP53* frequency is latitude dependent, Shi et al. have found that rs1042522 *TP53* polymorphism rather associates with winter temperature [23].

The rs17878362 is a 16-base pair (bp) insertion/duplication in intron 3 of *TP53* gene consisting of one copy (A1 allele) or two copies (A2 allele) of the sequence ACCTGGAGGGCTGGGG. Gemignani et al. have found that reduced levels of *TP53* mRNA are associated with the A2 allele [8]. It has not escaped of our notice that our study is a first association study of rs17878362 *TP53* polymorphism and rs1042522/rs17878362 *TP53* haplotypes with the risk of keloid scarring. No significant differences in the frequency distribution of both rs17878362 *TP53* alleles and genotypes have been found between keloid patients and controls in our study. The prevalence of A2 *TP53* allele in Polish newborns (14%) is also very close to the variant frequencies found in other populations of Slavic descent (15.5% and 17.0% in Czechs, 16.8% in Slovacks, or 12.7% in Russians) [18–20, 24]. We found also that linkage disequilibrium between rs1042522 and rs17878362 *TP53* polymorphisms (*D*′ 0.667 and *r*^2^ 0.221) was very close to values in other Slavic populations (*D*′ 0.570 and *r*^2^ 0.00 9 in Russians or *D*′ 0.693 and *r*^2^ 0.220 in Czechs). No significant differences in the frequency distribution of both rs1042522/rs17878362 *TP53* haplotype and diplotype have been found between keloid patients and controls in our study. On the other hand, the frequencies of both “wild-type” haplotype H1 (p.Arg72-A2) and “wild-type” diplotype D1 (H1/H1) were respectively of 7% or 8% higher as compared with control subjects (76% versus 69% for H1 or 56% versus 48% for D1, respectively). Also, Naccarati et al. [18] and Vymetalkova et al. [19] reported very similar H1 frequency in healthy adults in Czech Republic (67.1% or 65.5%, respectively).

We are fully aware that the major limitation of our study is relatively low statistical power due to small sample size. Therefore, we computed the minimal sample size necessary to achieve 80% statistical power for the detection of required ORs. The calculation for rs1042522 *TP53* polymorphism was performed using Genetic Power Calculator [25] in different models of inheritance and under the assumptions of 0.09% keloid prevalence [26], minor allele frequency equal 28.5%, case-to-control ratio equal 0.86, and 5% type I error rate (*α*). The calculated number of cases was dependent on the genetic model and varied from 201 keloid patients for the allelic test to 392 keloid ones for dominant model of inheritance.

## 5. Conclusion

Our results suggest the lack of association of rs1042522 and rs17878362 *TP53* polymorphisms and their haplotypes or diplotypes with the susceptibility to keloid scarring in Polish patients.

## Figures and Tables

**Table 1:** Association analysis of two *TP53* gene polymorphisms with keloid in Caucasians.

Polymorphism^a^ (position)	Allele^b^ (1/2)	Cases 1/2 (%)	Controls 1/2 (%)	*p*	Cases (*n* = 86)			Controls (*n* = 100)			*p* ^c^	OR (95% CI)^d^
					11	12 (%)	22	11	12 (%)	22		
rs1042522 (chr. 17: 7579472)	G/C	137/35 (80/20)	143/57 (72/28)	0.690	54 (63)	29 (34)	3 (3)	51 (51)	41 (41)	8 (8)	0.070	0.63 (0.39–1.04)
rs17878362 (chr. 17: 7579690)	A1/A2	152/20 (88/12)	172/28 (86/14)	0.496	67 (78)	18 (21)	1 (1)	73 (73)	26 (26)	1 (1)	0.485	0.80 (0.42–1.51)

^a^SNP position was indexed to the NCBI build 37 (GRCh37.p13). ^b^Allele 1 and allele 2 were defined as the nonsusceptible allele or the risk allele, respectively. ^c^*p* values for logistic regression in additive mode of inheritance of the risk allele. ^d^ORs and CIs were calculated using the nonsusceptible allele as a reference.

**Table 2:** Association analysis of *TP53* haplotypes with keloid in Caucasians.

Haplotype	rs1042522 (G/C)	rs17878362 (A1/A2)	Keloid *n* (%)	Control *n* (%)	*p* ^a^
H1	G	A1	130 (76)	138 (69)	0.132
H2	G	A2	7 (4)	5 (2)	0.449
H3	C	A1	22 (13)	34 (17)	0.232
H4	C	A2	13 (7)	23 (12)	0.213

Global score statistics = 3.776, degrees of freedom = 3, *p*=0.287					

^a^ *p* values for haplotype-specific statistics.

**Table 3:** Association analysis of *TP53* diplotypes with keloid in Caucasians.

Diplotype	Haplotype 1	Haplotype 2	Keloid *n* (%)	Control *n* (%)	*p*	OR (95% CI)
D1	H1	H1	48 (56)	48 (48)	—	—
D2	H1	H2	6 (7)	3 (3)	0.490	2.00 (0.47–8.46)
D3	H1	H3	18 (21)	20 (20)	0.783	0.90 (0.41–1.91)
D4	H1	H4	11 (13)	20 (20)	0.159	0.55 (0.24–1.27)
D5	H2	H2	—	—	—	—
D6	H2	H3	—	—	—	—
D7	H2	H4	—	1 (1)	—	—
D8	H3	H3	1 (1)	5 (5)	0.207	0.20 (0.02–1.78)
D9	H3	H4	1 (1)	3 (3)	0.618	0.33 (0.03–3.20)
D10	H4	H4	1 (1)	—	—	—

## Data Availability

The data used to support the findings of our study are included within the article.

## Supplementary Materials

Supplementary Materials Supplemental Table 1. Clinical characteristics of the keloid patients (*n* = 86).
