# Supplementary material for: Two Functional TP53 Genetic Variants and Predisposition to Keloid Scarring in Caucasians
Source: Dermatol Res Pract. 2019 Nov 13;2019:6179063. doi: 10.1155/2019/6179063 (PMC6881576; doi:10.1155/2019/6179063)
Supplement: Supplementary Materials — Supplemental Table 1. Clinical characteristics of the keloid patients (n = 86). [file 6179063.f1.pdf]

Supplemental Table 1. Clinical characteristics of the keloid patients (n=86).

Variable	Keloid patients n (%)
Family history of keloid (first degree relative)	10 (11.6)
Females	69 (80.2)
Multiple keloid scars	26 (30.2)
Etiology of keloid scars:	
Postoperative complication	47 (54.6)
Posttraumatic complication	9 (10.5)
Burning	9 (10.5)
Piercing ear	5 (5.8)
Vaccination	2 (2.3)
Insect bite	2 (2.3)
Unknown	12 (14.0)
Location of keloid scars:	
Head and neck	28 (26.4)
Chest	23 (21.7)
Back	10 (9.4)
Abdomen	13 (12.3)
Extremities	32 (30.2)
Total *	106 (100.0)

* Due to multilocality of keloid scars in some patients, number of keloid location is higher than the number of studied subjects.
